# Development of a GnRH-PGF_2α_ Based Synchronization and Superstimulation Protocol for Fixed-Time Mating in Llama Embryo Donors

**DOI:** 10.3389/fvets.2020.595889

**Published:** 2020-11-12

**Authors:** Enzo German Zampini, Maria Fernanda Veiga, Fernanda Gabriela Fumuso, Luciana Cabido, Deborah Margarita Neild, Maria Graciela Chaves, Marcelo Horacio Miragaya, Virginia Luz Trasorras

**Affiliations:** ^1^Universidad de Buenos Aires, Facultad de Ciencias Veterinarias, Instituto de Investigación y Tecnología en Reproducción Animal, Cátedra de Teriogenología, Buenos Aires, Argentina; ^2^Consejo Nacional de Investigaciones Científicas y Técnicas, Buenos Aires, Argentina; ^3^Dirección Provincial de Desarrollo Ganadero, Gobierno de la Provincia de Jujuy, Jujuy, Argentina

**Keywords:** camelids, follicular wave, ovarian dynamic, embryo transfer, ovarian superstimulation

## Abstract

The aim of the present study was to evaluate the application of a GnRH-PGF_2α_ based synchronization and superstimulation protocol for fixed-time natural mating in llama embryo donors. All females (*n* = 8) received 8 μg IM of GnRH analog (GnRHa; buserelin) on day 0, regardless of follicular status. After eight days, another GnRHa dose was administered followed by 250 μg IM PGF_2α_ (cloprostenol). A dose of 1000 IU IM of equine chorionic gonadotrophin (eCG) was applied on day 12 and a new dose of PGF_2α_ was administered on day 13. All embryo donors were mated with a male of proven fertility followed by a GnRHa dose on day 18. 24 h later, mating was repeated with a different male. Transcervical uterine flushing for embryo recovery was carried out on all females on day 26. Recipient females received one dose of GnRHa (day 0) two days after the first mating of embryo donor females. A 75% (6/8) of embryo donors responded to the superstimulation treatment with a range of 2 to 5 corpus luteums (CLs) on embryo recovery day. A total of 24 CLs were registered, with a mean of 4 ± 0.9 CLs per female. Embryo recovery rate was 66.7% (16/24), with a range of 0 to 4 embryos and a mean of 2.7 ± 1.5 embryos per female. Regarding quality of the recovered embryos, 56.2% were grade I, 6.2% were grade II and 37.5% were grade V (untransferable; arrested morulae). Grade I and II embryos (*n* = 10) were transcervically transferred into recipient females (*n* = 10) six days after inducing their ovulation. At 24 days after embryo transfer (ET), a 50% pregnancy rate was registered. In conclusion, a group of llama embryo donors can be synchronized and superstimulated using a fixed-time mating protocol based on GnRHa, PGF_2α_, and eCG without the necessity of using ultrasonography in the field.

## Introduction

Breeding llamas (*Lama glama*) is an economically relevant animal husbandry activity for a large sector of the high-altitude populations of South America, as the by-products of meat, fiber and manure are all put to use. Llamas are also employed for transporting cargo and represent an important cultural symbol for the region. Total llama population is estimated to be 5 million animals (1) in South America, concentrated mainly in Bolivia, Peru, and Argentina. Over the last few years, an increase in the consumption of llama meat has occurred because its palatability and low cholesterol content respond to current market demands, thus suggesting a favorable scenario for the production of these species. One of the existing problems llama producers face is the phenotypical expression of malformations (prognathism, conformation defects, heterochromatism, etc.), as a consequence of intense consanguinity in the herds due to internal replacement and mating of closely related animals. On the other hand, although this species presents a high capacity for adaptation to different adverse environmental conditions, conception and embryo mortality rates are highly variable under natural conditions (2). The mean annual fertility (birthing rate) in alpacas (*Vicugna pacos*) and llamas in South America can be as low as 45% (3). This low reproductive performance has different causes, for example, a late puberty (4), short reproductive seasonality in their natural habitat (5), long gestations (335 to 360 days) and birthing only one offspring at a time (6–8). Conventional reproductive management currently undertaken by most llama producers, consists in observing the sexual behavior of the females when confronted with a male and indicating natural mating when receptivity is detected, this being a labor-intense activity which demands a considerable amount of time (9). However, it is known that conception rates are lower (50–75%) when the females are mated based on their sexual behavior rather than when mating is indicated in the presence of a dominant growing follicle detected by ultrasonography (10, 11). Therefore, the establishment of a protocol that allows the presence of one or more dominant follicles at a fixed time, would not only make it possible to achieve better pregnancy rates, but would also facilitate the practices of herd management (12). In addition, applying ovarian superstimulation treatments combined with embryo transfer (ET) in genetically valuable animals, would make it possible to obtain a greater number of offspring per female per year, thus maximizing their reproductive efficiency (13, 14). In this way, the generation interval would be shortened, and genetic progress would accelerate (15).

The objective of this study was to evaluate the efficiency of a simple, fixed-time synchronization-ovarian superstimulation protocol in llamas, for use in an embryo transfer program, that does not depend on ultrasonography and is based on the administration of two doses of a GnRH analog, prostaglandin and equine chorionic gonadotrophin.

## Materials and Methods

### Environmental Conditions

This study was carried out during the month of May in the La Intermedia establishment in the town of Abra Pampa, located in the Department of Cochinoca of the Province of Jujuy, Argentina. The area is an Andean plateau situated 3,480 meters above sea level (22° 38' S−65° 69' W). This area corresponds to the Ecoregion of the dry Puna, with rainfall that varies between 100 and 400 mm per year, concentrated mainly between November and March (250 to 350 mm), a time of year when maximum temperatures reach 25°C. Winter is dry, with maximum temperatures in the region of 20°C, being warm during the day and fresh at night.

### Animals

A total of 48 non-gestating and non-lactating females, between 2 and 8 years old, with a body condition score of 3 (body condition score from 1 = thin to 5 = obese) were used in this study. They were kept separate from the group of males and were fed with natural pastures and had free access to water.

### Experimental Design

#### Treatment in Female Donors

**Figure 1:**
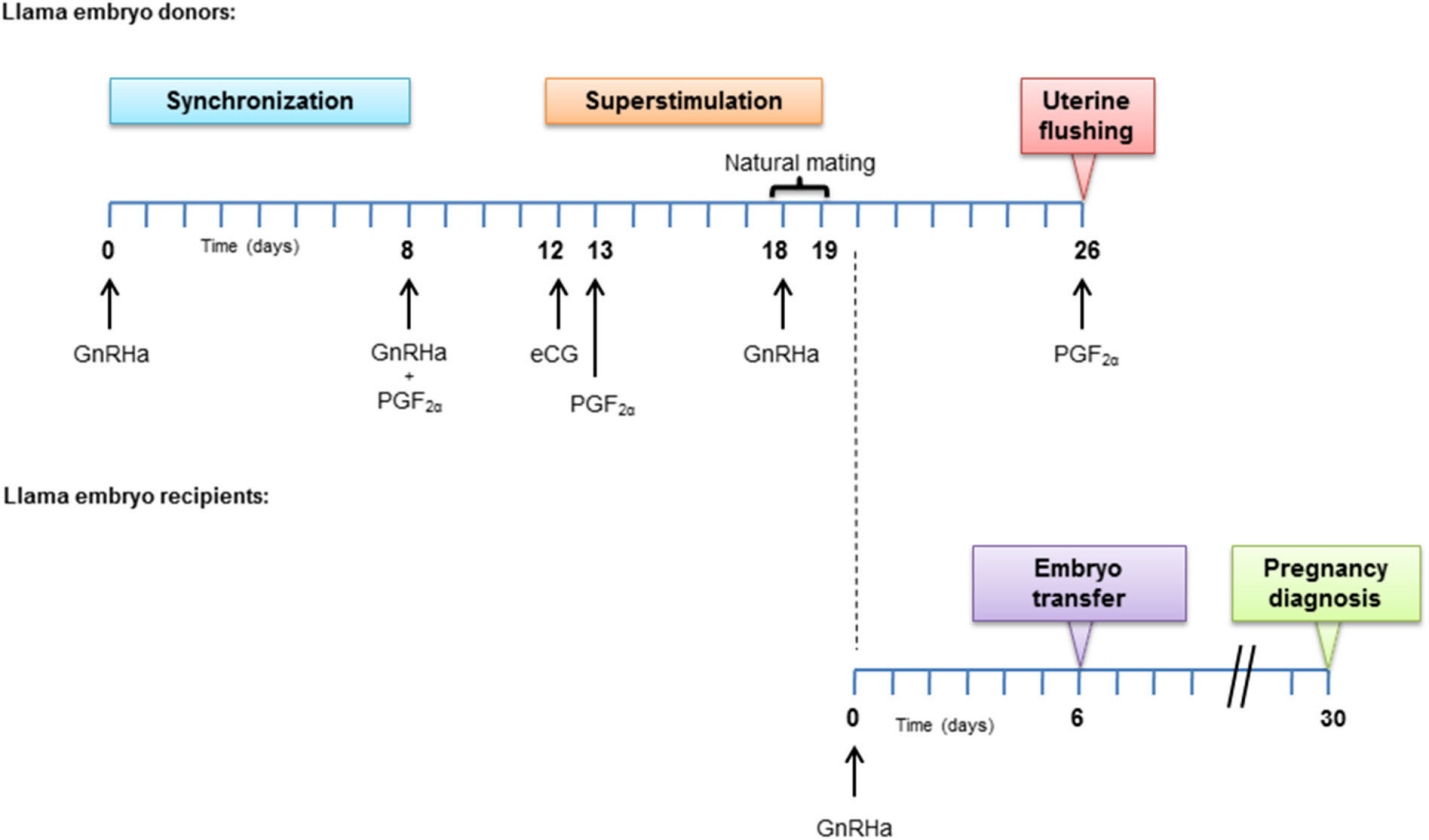
Experimental design. Hormone treatment applied in embryo donors and recipient females.

The complete experimental design is described in Figure 1. On day 0, all donor females (*n* = 8) received 8 μg IM of GnRH analog (GnRHa; buserelin acetate; Receptal®, Intervet, Buenos Aires, Argentina) for inducing endogenous LH release. Eight days later, another GnRHa dose was administered followed by 250 μg IM of PGF_2α_ (cloprostenol; Ciclase DL®, Syntex S.A., Buenos Aires, Argentina). On day 12 an IM dose of 1,000 IU of equine chorionic gonadotrophin (eCG; Novormon®, Syntex, Argentina) was injected (16). On day 13, another dose of PGF_2α_ was administered and 5 days later (day 18) natural mating with a male *Lama glama* of proven fertility was indicated for each female (*n* = 6). After natural mating, all females received a dose of GnRHa and 24 h later, natural mating was repeated with a different male, with the objective of minimizing the male effect.

#### Embryo Recovery and Evaluation

Uterine flushing was carried out non-surgically for embryo recovery, 8 days after the first mating (17). The maneuvers were carried out with the female either standing or in sternal recumbency. The animal was restrained in stocks, the tail was wrapped, and the rectum was emptied of feces. The perineum was then scrubbed using a hypoallergenic detergent, rinsed carefully with clean water and then dried. Restless females received 0.2 mg/kg IV xylazine (Xilazina® 10%, PRO-SER S.A., Buenos Aires, Argentina) before flushing. A Foley catheter (12 or 16 Fr, according to female size), with a stylet inserted into the catheter to keep it from bending during recto-vaginal manipulation, was used. Uterine flushing was done by placing the catheter cuff cranial to the internal cervical os and insufflating it with 5 or 10 ml of air (according to catheter gauge). The whole uterus was flushed 4 to 5 times using Ringer-Lactate solution, previously warmed (30-35°C), with a total volume of 500 ml. The recovered medium was filtered through a 70 μm EmCon^TM^ filter for embryos. After the flushing, donor females received a single IM dose of PGF_2α_ to induce luteolysis. The fluid in the flushing filter was placed in warmed reticulated Petri dishes and embryos were identified using a stereomicroscope. Embryos were classified according to their morphology following the criteria set by Tibary and Anouassi (18), using a grade scale from 1 to 5. Only morphologically normal (grade 1 and 2) blastocyst stage embryos were transferred to recipient females.

#### Management of the Recipient Females

All recipient females (*n* = 40) received 8 μg IM of GnRHa two days after the first natural service of the embryo donors (recipients' day 0). Transcervical ET was carried out on day 6 after GnRHa administration (17). The preparation of recipient females was as for donor females. A lubricated gloved hand was inserted in the rectum to hold the cervix while an assistant separated the vulva labia and an ET pipette, covered with a sterile sheath (IMV®, France) and carrying the 0.25 ml straw (IMV®, France) containing a single embryo, was inserted into the vagina. Cervical threading was performed aided by transrectal manipulation and the embryo was deposited in the uterine horn ipsilateral to the CL (17).

##### Pregnancy Diagnosis

Pregnancy diagnosis was carried out by transrectal ultrasonography, observing an embryonic vesicle 24 days after ET (corresponding to pregnancies of 32 days).

## Results

Donor females were evaluated by transrectal ultrasonography before carrying out the uterine flushing on day 26. Of the 8 females used, 75% (6/8) responded to the ovarian superstimulation treatment with a range of 2 to 5 CLs. A total of 24 CLs were registered, with a mean of 4 ± 0.9 CLs per female, and an occurrence of 58.3% in the left ovary (LO; 2.3 ± 0.5) and 41.7% in the right ovary (RO; 1.7 ± 1). A 25% (2/8) of the females presented a large anovulatory luteinized follicle.

**Figure 2:**
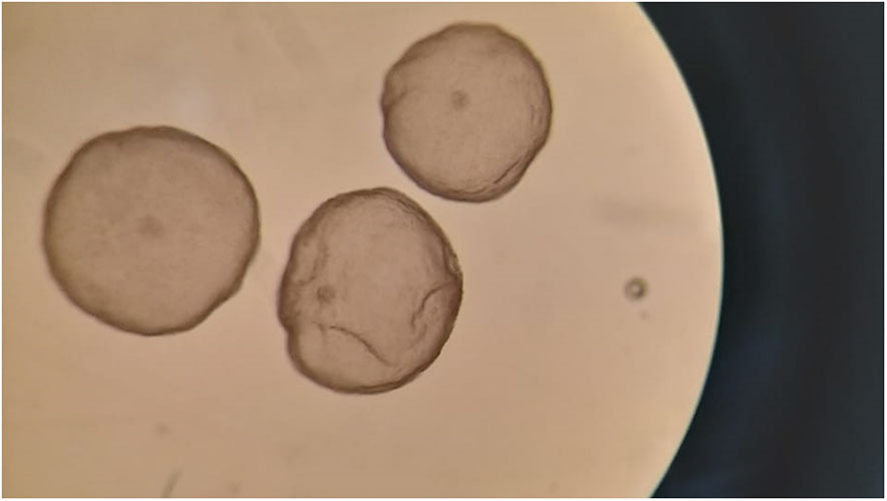
Hatched blastocysts (grade I) obtained 8 days after natural mating of an ovarian-superstimulated female llama.

A total of 16 embryos were recovered from the 6 donor females that were flushed (range: 0 to 4 embryos per animal), with a mean of 2.7 ± 1.5 embryos recovered per female (Figure 2). Embryo recovery rate (number of embryos recovered / number of CLs observed) was 66.7% (16/24). Of the recovered embryos, 56.2% (9/16) were grade I, 6.2% (1/16) grade II and the remaining 37.5% (6/16) were grade V (non-transferrable; arrested morulae).

Only grade I and II hatched blastocysts (*n* = 10) were transferred to the uterus of recipient females. An 80% (8/10) of the recipient llamas presented a CL in the left ovary and a 20% (2/10) in the right ovary.

The pregnancy rate was 50% (5/10) twenty four days after ET (3 pregnancies with a CL in the LO and 2 pregnancies with a CL in the RO) and pregnancies were again confirmed 80 days post ET.

## Discussion

The protocol evaluated in the present study was efficient in superstimulating embryo donor llamas without the need for ultrasonography. Ovarian response to the induction of multiple follicle growth in llamas is extremely variable, ranging between 0 and 17 ovulations or CLs and a recovery of 0 to 4 transferrable embryos per animal (13). One of the causes of this high variability in response is the stage of ovarian dynamics at the beginning of the superstimulatory treatment as the presence of a dominant follicle induces atresia in the rest of the follicles of that same wave (19). According to Miragaya et al. (20), beginning ovarian superstimulatory treatment in the presence of a follicle >5 mm in diameter induces the growth of only that follicle. To accomplish this latter objective, a natural luteal phase can be generated by inducing ovulation of the dominant follicle, with the resulting formation of a functional CL. In this manner, the inhibitory effect on follicular activity of progesterone produced by the CL is achieved (21). There are some protocols to induce multiple follicular growth in llamas based on the application of a single dose (1000 IU) of eCG, at different days after administering GnRH (22–24) or LH (25, 26) to induce ovulation of the dominant growing follicle previously monitored by ultrasound. In the current study, 75% of the llamas responded positively to the superstimulation protocol with a mean 4 CLs per female. These results were superior to those obtained by Bourke et al. (23), who achieved a 47% response to treatment, with an average of 2.4 ± 0.55 CLs per animal. In this last study, multiple follicular development was stimulated with eCG seven days after inducing ovulation with GnRH. It is possible that another dominant follicle could have already developed at the time of applying the ovarian superstimulatory treatment, because a new follicular wave emerges on average 2 to 3 days after inducing ovulation (9, 27). In addition, according to Ferrer et al. (28), follicle diameter is maintained below 7 mm until 6 days after induction. It is probable that the presence of this dominant follicle has an inhibitory effect on the development of the new follicles, thus reducing the response to ovarian superstimulatory treatment. In the Bourke et al. study (23), 1.4 ± 0.45 embryos were obtained per female, a lower result than the 2.7 ± 1.5 embryos recovered in the present study. These authors used 19 females, 14 of which received a single natural mating, and the remaining 5 received two matings separated by 24 h, obtaining an embryo recovery rate of 22 and 72%, respectively. They attributed the low results to the implementation of a single natural mating for each superstimulated female. Other studies have used the same ovarian superstimulation treatment, two days after administering GnRH in the presence of a dominant follicle, registering in the case of Vásquez (24), a mean of 6.5 ± 3.1 CLs/female and recovering 4 ± 2.6 embryos/female, while Aller et al. (22) observed 5.5 ± 3.1 CLs/female and obtained 2.1 ± 2.2 embryos/female. The embryo recovery rate achieved by Vásquez (24) was similar to that obtained in our study (65.3 vs. 66.7% respectively), both of which were superior to the rate of 40.7% obtained by Aller et al. (22). Based on inducing ovulation with LH, Huanca et al. (26) registered a higher mean of CLs (10.1 ± 2.9 CLs/animal) at the time of embryo recovery after administering eCG two days after LH. Evangelista et al. (25) used this same protocol, but the ovarian superstimulatory treatment was applied three days after LH, obtaining 9.3 ± 3.4 CLs/female. In these two studies, embryo recovery rate was 48.3% (26) and 37.7% (25), and this last low percentage could be attributed to the single natural mating used for each superstimulated female donor. Implementing double natural mating with different males on embryo donors that have been subjected to an ovarian superstimulation treatment, not only improves embryo recovery rates (23), it also allows genetic diversity to be increased, as has been recently proven in alpacas (29). In this way, not only is genetic variability increased, but phenotypical expression of congenital malformations could be avoided.

In an ET program, not only is the number of embryos recovered important, but it is also essential to consider the embryo quality. In our study, 62.5% of the embryos recovered were good quality and in condition to be transferred. Perhaps, the 37.5% that were arrested at the morula stage could be attributed to superstimulation treatments producing an increase in the number of recruited follicles and accelerating their growth and development, hence possibly leading to the ovulation of oocytes with immature nuclei or cytoplasm, thus affecting their future fertilization and/or embryo development (30). Furthermore, according to Aller et al. (22), there is a high positive correlation (0.91) between the number of follicles present on the day of natural mating and the estrogen plasma concentration in superstimulated llamas, and, based on studies in cattle (31, 32), these estrogen plasma levels could generate a reduction in the secretory activity of the oviductal epithelial cells and alter normal fertilization and/or early embryo development. Although to date no studies confirm this same effect in camelids, 5 of the arrested morulae obtained came from females that presented an ovarian cyst on the day of uterine flushing. According to Huanca et al. (26), ovarian cysts could affect results in superstimulated llamas in one of two ways: if hormonally active, they could alter correct embryo development and/or transport along the oviduct or, they could physically impede the normal ovulatory mechanism for the rest of the follicles.

In all the above-mentioned superstimulation protocols, trained personnel and transrectal ultrasonography are required, as prior synchronization of follicular dynamics was not carried out in the embryo donors. Thus, these methods are not very practical for applying in the field. In our study, we were able to establish a simple protocol that controls follicular growth in a group of females and guarantees the presence of two or more dominant follicles at a fixed-time, without the need of previous reproductive evaluation. Although it would be important to increase the number of females treated, implementing this protocol could improve pregnancy rates, facilitate management and allow natural mating and/or artificial insemination to be programmed simultaneously in larger groups of animals. In addition to being used for ET programs, it could also be applied for obtaining embryos for cryopreservation and even to harvest oocytes in an ovum pick-up program.

## Conclusion

It is possible to implement a synchronization-superstimulation fixed-time protocol in llamas using two doses of a GnRH analog, with two doses of prostaglandin F_2_ alfa prior to eCG, without the need for ultrasonography. This would make field work much easier, gaining independence from the need for personnel trained in imaging diagnostics.

## Data Availability Statement

The raw data supporting the conclusions of this article will be made available by the authors, without undue reservation.

## Ethics Statement

The animal study was reviewed and approved by Comité Institucional de Cuidado y uso de Animales de Laboratorio (CICUAL).

## Author Contributions

VT and EZ contributed to the conception and design of the study. EZ, MV, FF, LC, and VT provided help with field work. EZ and VT wrote the manuscript. MC, DN, and MM made critical revisions to the paper and DN contributed to language editing. All authors read, edited and approved the final manuscript.

## Conflict of Interest

The authors declare that the research was conducted in the absence of any commercial or financial relationships that could be construed as a potential conflict of interest.
